# PET/MRI versus PET/CT in oncology: a prospective single-center study of 330 examinations focusing on implications for patient management and cost considerations

**DOI:** 10.1007/s00259-019-04452-y

**Published:** 2019-08-13

**Authors:** Marius E. Mayerhoefer, Helmut Prosch, Lucian Beer, Dietmar Tamandl, Thomas Beyer, Christoph Hoeller, Dominik Berzaczy, Markus Raderer, Matthias Preusser, Maximilian Hochmair, Barbara Kiesewetter, Christian Scheuba, Ahmed Ba-Ssalamah, Georgios Karanikas, Julia Kesselbacher, Gerald Prager, Karin Dieckmann, Stephan Polterauer, Michael Weber, Ivo Rausch, Bernhard Brauner, Harald Eidherr, Wolfgang Wadsak, Alexander R. Haug

**Affiliations:** 1 Department of Biomedical Imaging and Image-guided Therapy, Division of General and Pediatric Radiology, Medical University of Vienna, Waehringer Guertel 18-20, 1090 Vienna, Austria; 2 Department of Radiology, Memorial Sloan Kettering Cancer Center New York, New York City, NY USA; 3 Center for Medical Physics and Biomedical Engineering, Medical University of Vienna, Vienna, Austria; 4 Department of Dermatology, Medical University of Vienna, Vienna, Austria; 5 Department of Medicine I, Division of Oncology, Medical University of Vienna, Vienna, Austria; 6 Department of Respiratory and Critical Care Medicine and Ludwig Boltzmann Institute for COPD and Respiratory Epidemiology, Otto Wagner Hospital, Vienna, Austria; 7 Department of Surgery, Division of General Surgery, Medical University of Vienna, Vienna, Austria; 8 Department of Biomedical Imaging and Image-guided Therapy, Division of Nuclear Medicine, Medical University of Vienna, Vienna, Austria; 9 Department of Radiation Oncology, Medical University of Vienna, Vienna, Austria; 10 Department of Obstetrics and Gynecology, Medical University of Vienna, Vienna, Austria; 11 Siemens Healthineers, Siemens Healthcare Diagnostics GmbH, Vienna, Austria; 12 Center for Biomarker Research in Medicine–CBmed, Graz, Austria

**Keywords:** PET/MRI, PET/CT, Oncology, Patient management, Costs

## Abstract

**Purpose:**

PET/MRI has recently been introduced into clinical practice. We prospectively investigated the clinical impact of PET/MRI compared with PET/CT, in a mixed population of cancer patients, and performed an economic evaluation of PET/MRI.

**Methods:**

Cancer patients referred for routine staging or follow-up by PET/CT underwent consecutive PET/CT and PET/MRI, using single applications of [^18^F]FDG, [^68^Ga]Ga-DOTANOC, or [^18^F]FDOPA, depending on tumor histology. PET/MRI and PET/CT were rated separately, and lesions were assessed per anatomic region; based on regions, *per-examination* and *per-patient* accuracies were determined. A simulated, multidisciplinary team meeting served as reference standard and determined whether differences between PET/CT and PET/MRI affected patient management. The McNemar tests were used to compare accuracies, and incremental cost-effectiveness ratios (ICERs) for PET/MRI were calculated.

**Results:**

Two hundred sixty-three patients (330 same-day PET/CT and PET/MRI examinations) were included. PET/MRI was accurate in 319/330 examinations and PET/CT in 277/330 examinations; the respective accuracies of 97.3% and 83.9% differed significantly (*P* < 0.001). The additional findings on PET/MRI—mainly liver and brain metastases—had implications for patient management in 21/263 patients (8.0%). The per-examination cost was 596.97 EUR for PET/MRI and 405.95 EUR for PET/CT. ICERs for PET/MRI were 14.26 EUR per percent of diagnostic accuracy and 23.88 EUR per percent of correctly managed patients.

**Conclusions:**

PET/MRI enables more appropriate management than PET/CT in a nonnegligible fraction of cancer patients. Since the per-examination cost is about 50% higher for PET/MRI than for PET/CT, a histology-based triage of patients to either PET/MRI or PET/CT may be meaningful.

## Introduction

PET/MRI (positron emission tomography/magnetic resonance imaging) is a relatively novel hybrid imaging technique that has recently been introduced into routine clinical practice. Today, the vast majority of PET/MRI systems are installed in tertiary care centers, where this imaging technique is partly or mainly used for research [1]; worldwide, the number of PET/MRI systems is gradually increasing [1]. Compared with PET/CT (computed tomography)—the standard hybrid imaging technique—PET/MRI offers reduced radiation exposure and higher morphological soft-tissue contrast.

For oncologic imaging, several comparative studies between PET/MRI and PET/CT have been performed within the last couple of years, the majority in smaller-sized patient populations. Depending on the type of cancer investigated, the clinical setting, and the choice of PET radiotracer, these studies have either reported that PET/CT and PET/MRI perform equally well [2–13], or that PET/MRI has minor to moderate advantages [14–21]. It is questionable whether the latter results justify the use of PET/MRI in a routine setting, because the costs for purchase, installation, and maintenance of a PET/MRI system exceed those of PET/CT, and because the scan duration is typically longer with PET/MRI, which leads to a lower patient throughput [1]. In addition, clinical implications—such as changes in treatment strategy—of using PET/MRI instead of PET/CT have been documented in only a few studies [7, 17–19, 22].

The aim of this study was therefore to (1) prospectively investigate the clinical impact of PET/MRI, compared with PET/CT, in a mixed population of cancer patients, and to (2) perform an economic evaluation of PET/MRI through comparison with PET/CT, using clinically oriented cost-effectiveness analyses.

## Materials and methods

### Patients and design

All patients with histology-proven cancers who were referred to our institution for routine pretherapeutic staging or posttherapeutic follow-up by PET/CT, and who were eligible for participation according to the criteria below, were invited to participate in our prospective study. Approval from the Ethics Committee of the Medical University of Vienna and written informed consent from all patients were obtained. Exclusion criteria were pregnancy; inability to understand the study outline or give consent; age < 18 years; contraindications to MRI according to safety guidelines; previous adverse reactions to ionized or gadolinium-based contrast media; and, for patients scheduled to receive [^18^F]FDG (2-deoxy-2-[^18^F]fluoro-d-glucose) for PET, a blood glucose level > 150 mg/dL.

Enrolled patients first underwent PET/CT, and then, within 2 hours, PET/MRI, using a single radiotracer injection at the respective standard time point for that radiotracer (see below) for both examinations.

### PET radiotracers and dosage

For patients with well-differentiated neuroendocrine tumors (NET G1–2), PET/CT was performed 45–60 min after intravenous administration of 160–180 MBq of [^68^Ga]Ga-DOTANOC (conjugate of the somatostatin analogue 1-Nal3-octreotide and [^68^Ga]-labeled 1,4,7,10-tetraazacyclododecane-N,N′,N″,N‴-tetraacetic acid), synthesized as previously described [23]. For patients with medullary thyroid carcinoma (MTC), pheochromocytoma, or paraganglioma, PET/CT was performed 60 min after intravenous administration of 3 MBq/kg body weight of [^18^F]FDOPA (6-[^18^F]fluoro-3,4-dihydroxy-l-phenylalanine), commercially obtained from local vendors. For all other cancer patients, PET/CT was performed 60 min after intravenous administration of 3 MBq/kg of [^18^F]FDG, produced in-house.

### Imaging protocols

PET/CT was performed using a 64-row multidetector, hybrid PET/CT system (Biograph TruePoint TrueView 64; Siemens, Erlangen, Germany). The PET system offers an axial field-of-view of 216 mm, a sensitivity of 7.6 cps/kBq, and a transaxial resolution of 4–5 mm (measured according the NEMA NU2 protocol). PET imaging was performed at 4 min/bed position, and images were reconstructed using the point-spread function (PSF)-based reconstruction algorithm TrueX, with four iterations and 21 subsets, 5-mm slice thickness, and a 168 × 168 matrix size. Contrast-enhanced venous-phase CT was used for attenuation correction and was performed after the intravenous injection of 90–120 ml of a triiodinated, nonionic contrast medium at a rate of 4 ml/s, with a reference tube current of 230 mAs (with tube current modulation), a tube voltage of 120 kVp, a collimation of 64 × 0.6 mm, a 5-mm slice thickness with a 3-mm increment, and a 512 × 512 matrix. In addition, arterial phase CT of the upper abdomen (i.e., from the diaphragm to the lower pole of the kidneys) was acquired for all cancers except lymphoma, myeloma, and nonsmall cell lung cancer.

PET/MRI, covering the same anatomy as PET/CT, was performed directly after PET/CT, using a fully integrated PET/MR system (Biograph mMR; Siemens, Erlangen, Germany) operating at 3 T, with high-performance gradient systems (45 mT/m) and a slew rate of 200 T/m/s, and equipped with a phased-array body coil. The PET system offers an axial FOV of 256 mm, a sensitivity of 13.2 cps/kBq, and a transaxial resolution of 4.4 mm (measured according the NEMA NU2 protocol). PET imaging was performed at 100–150 min post original tracer administration, at 5 min/bed position, and images were reconstructed using the PSF-based algorithm HD-PET, with three iterations and 21 subsets, a 4.2-mm slice thickness, and a 172 × 172 matrix size. For all cancer patients, the following two pulse sequences were obtained for the entire anatomy: (1) an axial, two-point Dixon, three-dimensional, volume-interpolated, T1-weighted (T1w) breath-hold MR sequence (VIBE) for attenuation correction, with a repetition time (TR)/echo times (TE) of 3.6/TE1 = 1.23 ms, TE2 = 2.46 ms; one average, two echoes; a 10° flip angle; a 320 × 175 matrix with a 430 × 309 mm FOV; and a 3-mm slice thickness with 0.6-mm gap; and (2) a coronal, T2-weighted, HASTE (half-Fourier acquisition single-shot) turbo spin-echo sequence, with a TR/TE of 1400/121 ms; a 160° flip angle; a 256 × 256 matrix with a 380 × 380 mm FOV; and a 6-mm slice thickness with a 1.2-mm gap. Depending on the cancer type, MR pulse sequences listed in Table 1 were added, based on the standard MR sequence protocols used for stand-alone MRI at our institution.

**Table 1:** PET/MRI protocol: additional MRI sequences for different types of cancer

	Axial 2-point Dixon T1 VIBE 3D	Axial EPI SPAIR DWI free-breathing	Dynamic Gd-enhanced T1 VIBE with fat saturation	Sagittal T1 TSE (spine only)
TR (ms)	4.02/1.23	6800	4.56	610
TE (ms)	2.46	63	2.03	9.6
Flip angle (°)	10	180	9	150
Field of view (mm)	296 × 430	168 × 104	380 × 309	320 × 100
Matrix size	154 × 320	440 × 340	195 × 320	320 × 75
Slice thickness (mm)	3 + 0.6 gap	6 + 1.2 gap	3 + 0.6 gap	3 + 1.5 gap
Other parameters	–	b50, b800; ADC maps	0.025 mmol/kg of Gd-EOB-DTPA or 0.1 mmol/kg of an extracellular Gd-based agent	–
Cancer types	Lymphoma, myeloma, and CUP	Lymphoma, myeloma, and CUP	All cancers except lymphoma, myeloma, and CUP	Myeloma

### Image analysis

A senior board-certified radiologist and a senior board-certified nuclear medicine physician rated PET/CT, and 2 weeks later, PET/MRI examinations, in consensus, side-by-side, blinded to the patients’ reports from clinical practice, and the respective other technique (PET/CT or PET/MRI), in random order.

Separately for PET/MRI and PET/CT, raters had to decide which of the following 19 organs/tissues were positive for malignant lesions, based on pathological PET tracer accumulations and/or morphological CT/MRI features: brains; thyroid; left and right lung/pleura; left and right liver lobe; spleen; pancreas; esophagus/stomach; small bowel; large bowel/rectum; uterus/cervix/ovaries; left and right kidney; left and right adrenal gland; osseous structures; soft tissues (skin/muscle/fat); and other organs. In addition, the following 12 lymph node stations were assessed: right and left cervical (including supraclavicular, occipital, and preauricular nodes); right and left infraclavicular/axillary; mediastinal; hilar; retroperitoneal/periaortic; mesenteric; right and left pelvic; and right and left inguinal. Lesion numbers within each of these 31 anatomic regions (19 organs/tissues and 12 lymph node stations) were recorded, with a maximum of ten.

### Clinical impact/simulated multidisciplinary team meeting

Following their independent evaluation, PET/CT and PET/MRI findings were compared. A simulated multidisciplinary team (MDT) meeting, consisting of the two raters from radiology and nuclear medicine, two oncologists, a dermatologist specializing in skin cancer, two surgeons, and a radiation oncologist (all board-certified), reviewed all clinical, histological, laboratory, and imaging data. The MDT verified findings using a composite reference standard that relied on previous and follow-up CT, MRI, PET/CT, and PET/MRI (i.e., on lesion progression or regression under therapy, or new lesion formation), and, if clinically indicated, biopsies. Furthermore, the MDT made the following decisions on a *per-examination* basis:

1. Involvement of additional anatomic regions *with* implications for clinical management or therapy, visible exclusively on *either* PET/MRI *or* PET/CT
2. Involvement of additional anatomic regions *without* implications for clinical management or therapy, visible exclusively on *either* PET/MRI *or* PET/CT
3. *Additional lesions* in one or more involved anatomic regions, *with* implications for clinical management or therapy, visible exclusively on *either* PET/MRI *or* PET/CT
4. *Additional lesions* in one or more involved anatomic regions, *without* implications for clinical management or therapy, visible exclusively on *either* PET/MRI *or* PET/CT
5. Equivocal findings on *either* PET/MRI *or* PET/CT, *with* implications for clinical management

For patients who had undergone more than one same-day PET/CT and PET/MRI within the course of this study (i.e., patients who were examined with PET/CT and PET/MRI for pretherapeutic staging, and then again at one or more time points for restaging after therapy), changes in management or therapy, due to differences between the two imaging techniques in terms of involved regions or lesion numbers, were counted only once, unless they were due to new lesion formation in the time interval between the different time points.

### Statistical and economic analysis

Region-based involvement on PET/MRI and PET/CT was used to calculate examination-based accuracies for the two imaging techniques. A test was considered accurate if the number of involved regions and the number of lesions per involved region were correctly assessed, compared with the reference standard, regardless of a possible clinical impact. The McNemar tests were then used to assess significant, examination-based differences between PET/MRI and PET/CT accuracies and clinical impact.

For the economic comparison between PET/MRI and PET/CT, two measures of effectiveness were used: (1) the percentage of accurate diagnoses and (2) the percentage of changes in clinical management, relative to the other test. Per-examination costs (in EUR), and the respective difference in costs between PET/MRI and PET/CT, were based on total cost of ownership, which included investment cost for the system (as supplied by the manufacturer) and maintenance costs (based on the maintenance contract) and number of examinations per year (using our institution’s standard of eight PET/MRI examinations/day and 13 PET/CT examinations/day). Costs for the cyclotron, PET radiotracer production equipment, and personnel (e.g., physicians and technicians) were not considered, because these are identical for PET/MRI and PET/CT. Straight line depreciation over 10 years was used to calculate the yearly asset’s loss of value for the two systems.

The following incremental cost-effectiveness ratios (ICERs) for PET/MRI were calculated:

1. ICER-1 = (cost(PET/MRI) − cost(PET/CT))/(accuracy(PET/MRI) − accuracy(PET/CT))
2. ICER-2 = (cost(PET/MRI) − cost(PET/CT))/(percentage of management changes(PET/MRI) − percentage of management changes(PET/CT))

The specified level of significance was *P* ≤ 0.05 for all tests. All statistical tests were performed using SPSS 24.0 (IBM Corp., Armonk, NY, USA).

## Results

### Patient characteristics

Between March 2014 and October 2017, 263 patients (111 women and 152 men; mean age, 56.4 ± 16.1 years; age range, 18–87 years) were enrolled. Cancer types and their absolute and relative frequencies are listed in Table 2. Same-day PET/CT and PET/MRI was performed once in 221 patients; twice in 27 patients; at three time points in ten patients; at four time points in five patients; and at five time points in one patient. Thus, a total of 330 same-day PET/CT and PET/MRI examinations (staging, 169; restaging, 161) were available for comparison.

**Table 2:** Absolute and relative frequencies of cancer types in 263 patients and 330 same-day PET/CT and PET/MRI examinations

Cancer type	Patients		Same-day examinations	
	*n*	%	*n*	%
Lymphoma (Hodgkin/NHL)	52	19.8	61	18.5
Nonsmall cell lung cancer	46	17.5	75	22.7
Neuroendocrine tumors (G1–2)	35	13.3	39	11.8
Melanoma	26	9.9	48	14.5
Pancreatic adenocarcinoma	16	6.1	17	5.2
Cancer of unknown primary	13	4.9	14	4.2
Multiple myeloma	11	4.2	11	3.3
Gynecological cancer	9	3.4	9	2.7
Colorectal cancer	9	3.4	9	2.7
Head/neck cancer	8	3.0	8	2.4
Sarcoma	7	2.7	8	2.4
Esophageal cancer	6	2.3	6	1.8
Breast cancer	6	2.3	6	1.8
Thyroid carcinoma (excl. MTC)	5	1.9	5	1.5
Cholangiocellular carcinoma	3	1.1	3	0.9
MTC	2	0.8	2	0.6
Skin squamous cell carcinoma	2	0.8	2	0.6
Renal cell cancer (clear cell)	1	0.4	1	0.3
Adrenal adenocarcinoma	1	0.4	1	0.3
Hepatocellular carcinoma	1	0.4	1	0.3
Gastric cancer	1	0.4	1	0.3
Gastrointestinal stroma tumor	1	0.4	1	0.3
Pheochromocytoma	1	0.4	1	0.3
Urothelial carcinoma	1	0.4	1	0.3

*NHL* non-Hodgkin lymphoma, *MTC* medullary thyroid carcinoma

Contrast media-enhanced MRI sequences were used in 187/263 patients (71.1%) and 244/330 same-day PET/MRI and PET/CT examinations (73.9%). Gd-EOB-DTPA was used in 49/263 patients (18.6%; 30 NETs, nine pancreatic adenocarcinomas, six colorectal cancers, three cholangiocellular cancers, and one hepatocellular cancer) and extracellular Gd-based agents in the remaining 138 patients (52.5%).

### Accuracy and implications for management

PET/MRI and PET/CT showed perfect agreement (i.e., same number of involved anatomic regions, same number of lesions per involved region) with each other, as well as with the reference standard (MDT) in 270/330 examinations (81.8%). Of the remaining 60 examinations (18.2%) with differences between PET/CT and PET/MRI, PET/MRI was accurate in 51, PET/CT in seven, and neither scan in two examinations (see below), relative to the reference standard. The respective examination-based accuracies for PET/MRI (97.3%) and PET/CT (83.9%) differed significantly (*P* < 0.001).

**Fig. 1:**
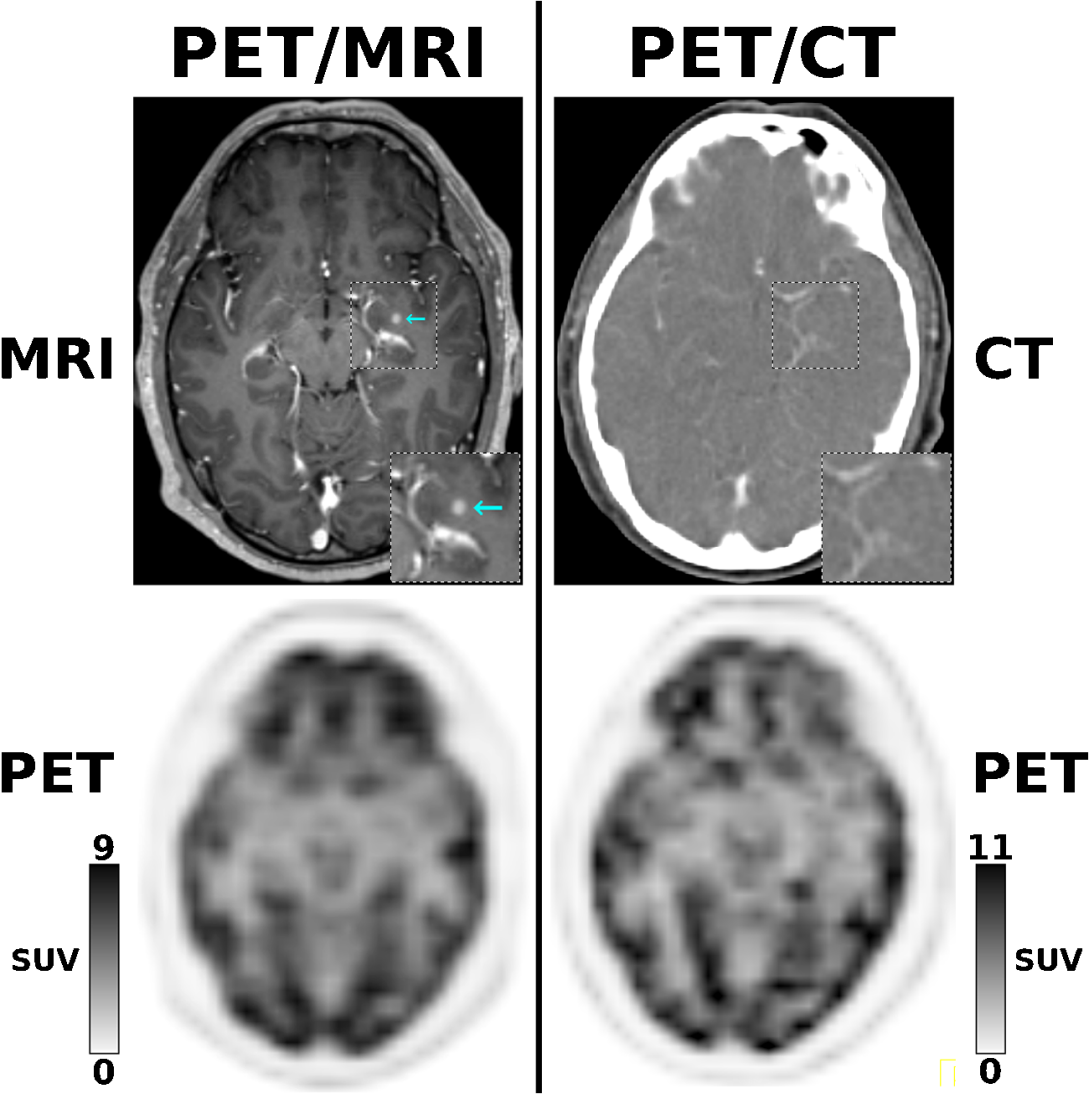
A 61-year-old patient with NSCLC stage IV, referred for staging before nivolumab treatment. While [^18^F]FDG-PET is unremarkable for both PET/MRI and PET/CT, the contrast-enhanced MRI component of PET/MRI depicts a small brain metastases in the left hippocampus (cyan arrow) that is not visualized on the contrast-enhanced CT component of PET/CT (× 1.5 magnifications in right lower corners), and for which radiation therapy is indicated

**Fig. 2:**
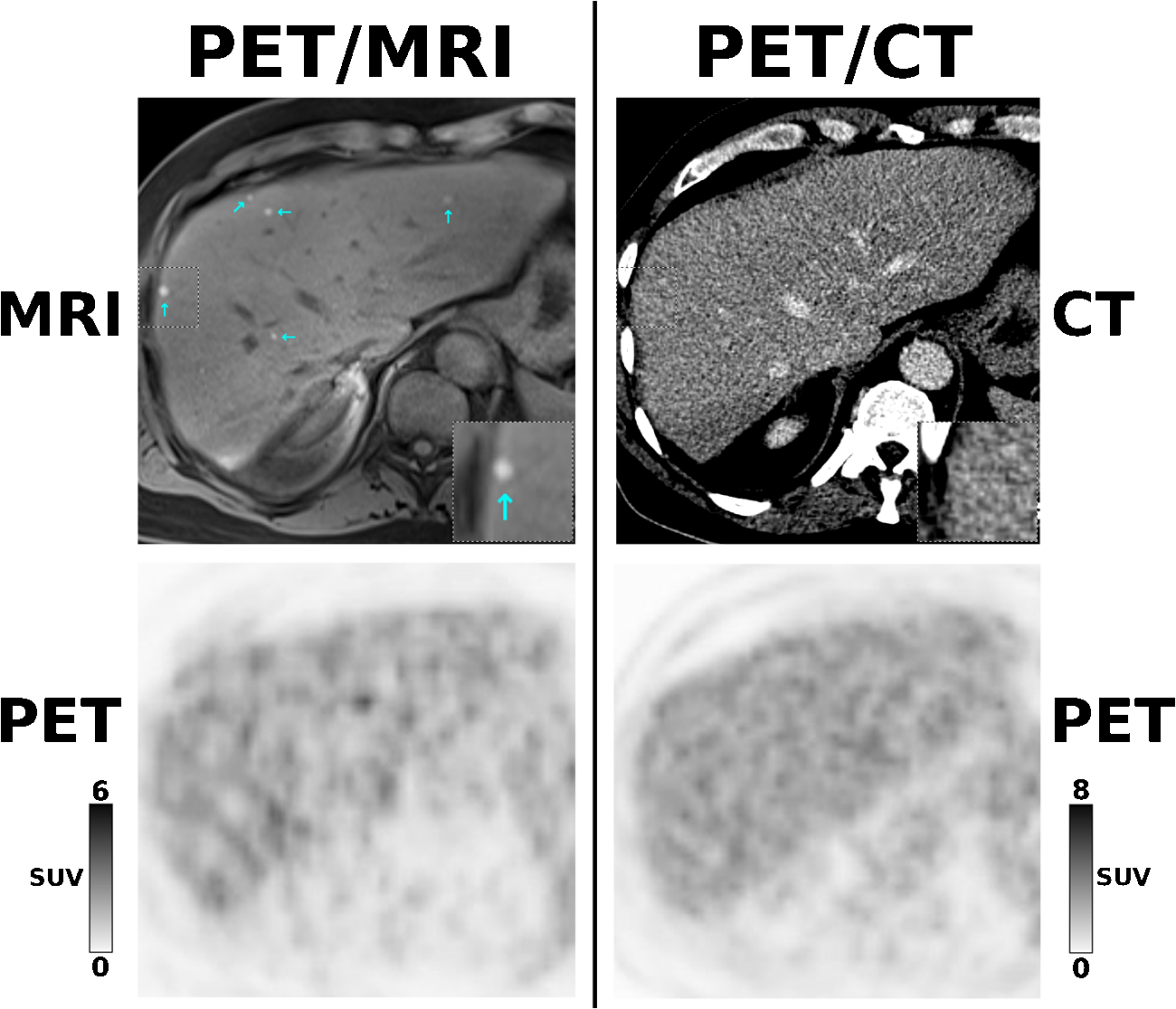
A 59-year-old patient with malignant melanoma stage IV, referred for follow-up after ipilimumab treatment. While [^18^F]FDG-PET is unremarkable for both PET/MRI and PET/CT, the contrast-enhanced MRI component of PET/MRI depicts multiple, newly developed small liver metastases (cyan arrows) that are not visualized by the contrast-enhanced CT component of PET/CT (× 2 magnifications of the segment VIII lesion in right lower corners). This changed the diagnosis to progressive disease and led to a switch from ipilimumab to PD1 antibody treatment

In 53 examinations, there were additional findings on the MRI component of PET/MRI that were not seen on PET/CT. These additional findings had implications for clinical management or therapy in 23/330 examinations (7.0%)—in 16 due to involvement of additional anatomic regions (see Figs. 1 and 2), in six due to additional lesions in at least a single region, and in one case because PET/CT findings were equivocal. However, none of the additional findings that were observed on nine PET/CT examinations, but not on PET/MRI—all of which were lung metastases seen on the CT component—had implications for clinical management or therapy. Notably, in two examinations, neither PET/MRI nor PET/CT were accurate, because PET/CT showed more lung lesions than PET/MRI, whereas PET/MRI showed involvement of the liver that was not visualized by PET/CT; here, despite not being accurate overall, PET/MRI had implications for clinical management or therapy, compared with PET/CT. Based on these data, and considering that some patients had undergone same-day PET/CT and PET/MRI at more than one time point, PET/MRI led to changes in clinical management or therapy in 21/263 patients (8.0%) (see Table 3).

**Table 3:** Changes in management due to additional findings on PET/MRI

Patient no.	Cancer type	Staging/restaging	Additional findings on PET/MRI	Management change relative to PET/CT
15	NET	Staging	Metastases in left liver lobe not visible on [^68^Ga]Ga-DOTANOC-PET/CT	Liver surgery in addition to primary tumor surgery
22	NSCLC	Staging	More brain metastases than on [^18^F]FDG-PET/CT	No additional MRI of the brain needed for radiation therapy planning
27	Pancreatic adenocarcinoma	Staging	Metastases in both liver lobes not visible on [^18^F]FDG-PET/CT	Palliative chemotherapy only instead of primary tumor surgery and chemotherapy
63	Melanoma	Staging	Brain metastases not visible on [^18^F]FDG-PET/CT	Additional radiation therapy/no additional MRI of the brain needed
82	Melanoma	Staging	Brain metastases not visible on [^18^F]FDG-PET/CT	Additional radiation therapy / no additional MRI of the brain needed
88	Cervical cancer	Staging	No urinary bladder infiltration by primary tumor ([^18^F]FDG-PET/CT suggestive of bladder infiltration)	Surgery and chemotherapy instead of just chemotherapy
93	NET	Restaging	Metastasis in left liver lobe not visible on [^68^Ga]Ga-DOTANOC-PET/CT	Follow-up MRI examinations at 3–6-month intervals
100	Colorectal adenocarcinoma	Restaging	More metastases in both liver lobes than visible on [^18^F]FDG-PET/CT	Chemotherapy only vs. chemotherapy and liver surgery
106	Pancreatic adenocarcinoma	Staging	Metastases in right liver lobe not visible on [^18^F]FDG-PET/CT	Palliative chemotherapy only vs. primary tumor surgery and chemotherapy
110	Melanoma	Staging	More brain metastases than on [^18^F]FDG-PET/CT	No additional MRI of the brain needed for radiation therapy planning
115	Pancreatic adenocarcinoma	Staging	Metastases in both liver lobes not visible on [^18^F]FDG-PET/CT	Palliative chemotherapy only vs. primary tumor surgery and chemotherapy
119	NET	Restaging	Metastases in right liver lobe not visible on [^68^Ga]Ga-DOTANOC-PET/CT	Liver surgery
127	Melanoma	Staging	More metastases in left liver lobe than visible on [^18^F]FDG-PET/CT	No liver surgery due to multiple metastases
139	NSCLC	Staging	Brain metastasis not visible on [^18^F]FDG-PET/CT	Additional radiation therapy/no additional MRI of the brain needed
139	NSCLC	Restaging	New brain metastasis not visible on [^18^F]FDG-PET/CT	No additional MRI of the brain needed for radiation therapy planning
140	NSCLC	Staging	Brain metastasis not visible on [^18^F]FDG-PET/CT	Additional radiation therapy/no additional MRI of the brain needed
151	NSCLC	Restaging	Brain metastasis not visible on [^18^F]FDG-PET/CT	Additional radiation therapy/no additional MRI of the brain needed
160	NSCLC	Staging	Brain metastasis not visible on [^18^F]FDG-PET/CT	Additional radiation therapy/no additional MRI of the brain needed
160	NSCLC	Restaging	New brain metastases not visible on [^18^F]FDG-PET/CT	No additional MRI of the brain needed for radiation therapy planning
212	Melanoma	Restaging	Multiple metastases in both liver lobes instead of single metastasis in right liver lobe, as suggested by [^18^F]FDG-PET/CT	Therapy switch from ipilimumab to pembrolizumab due to progression instead of stable disease
256	NSCLC	Restaging	Brain metastasis not visible on [^18^F]FDG-PET/CT	Additional radiation therapy/no additional MRI of the brain needed
260	NSCLC	Staging	Brain metastasis not visible on [^18^F]FDG-PET/CT	Additional radiation therapy/no additional MRI of the brain needed
263	NSCLC	Staging	Equivocal adrenal gland lesion on [^18^F]FDG-PET/CT, diagnosed as fat-containing adenoma on chemical shift MRI	No additional MRI required to complete staging

### Cost-effectiveness of PET/MRI

The total cost of ownership for 10 years was calculated 11.94 million EUR for PET/MRI and 13.19 million EUR for PET/CT (see Table 3), with 20,000 (10 × 250 workdays/year × 8 examinations/day) PET/MRI and 32,500 (10 × 250 workdays/year × 13 examinations/day) PET/CT examinations. Based on these numbers, the per-examination cost was calculated as 596.97 EUR for PET/MRI and 405.95 EUR for PET/CT.

Based on the higher accuracy of PET/MRI (+ 13.4% compared with PET/CT), and the higher percentage of changes in patient management or therapy due to PET/MRI (+ 8% compared with PET/CT), the ICER-1 of PET/MRI was 14.26 EUR per percent of diagnostic accuracy, and ICER-2 was 23.88 EUR per percent of correctly managed patients.

## Discussion

The results of our prospective study suggest that PET/MRI provides additional clinical value in terms of changes to more appropriate management in 8% of cancer patients who undergo PET/CT in routine clinical practice. This percentage is lower than that in the largest study thus far: with regard to clinical impact, Catalano et al. reported a superiority of PET/MRI over PET/CT for 16% of cancer patients in a retrospective analysis [17]. This discrepancy may be explained by the differences in relative frequencies of cancer types between our study and theirs (e.g., NSCLC, 17% vs. 7%; melanoma, 10% vs. 3%; breast cancer, 2% vs. 26% of the entire cohorts, respectively). Unlike Catalano et al., we also used PET radiotracers other than [^18^F]FDG, namely, [^68^Ga]Ga-DOTANOC for well-differentiated NETs. Notably, patients with well-differentiated NETs, for which [^68^Ga]Ga-DOTA-peptide-PET/CT is the technique of choice [24, 25], and which accounted for 13% of our population, were not included by Catalano et al. [17]. While our results clearly do not justify a general replacement of PET/CT with PET/MRI, they do suggest that certain subgroups—such as advanced-stage NSCLC and melanoma (see Table 3)—could benefit from undergoing PET/MRI instead of PET/CT.

In our study, the overall superiority of PET/MRI over PET/CT in terms of diagnostic accuracy (+ 13%) was mainly due to the superior performance of PET/MRI for the detection of brain and liver metastases (see Table 3, Figs. 1 and 2), which has been documented in previous smaller-sized studies [2, 8, 10, 11]. Similarly, the superiority of PET/CT for the detection of lung lesions—another previously reported finding [2, 26–28]—was also confirmed in our study. While the additional brain and liver metastases detected exclusively by the MRI component of PET/MRI had implications for management in 19/21 patients (see Table 3), the additional lung lesions detected exclusively by the CT component of PET/CT, but not by PET/MRI, did not have implications for management in any patient.

Importantly, in NSCLC, it has been shown that treatment of early brain metastases, while still asymptomatic, is associated with better control of neurologic manifestations and longer survival [29], and hence, the American College of Chest Physicians recommends cranial MRI (preferred over cranial CT) for routine imaging of clinical stage III–IV NSCLC patients [30], a strategy comparable with that proposed by the European Society of Medical Oncology [31, 32]. Similar recommendations on the use of cranial MRI to detect brain metastases exist for melanoma in stages III–IV, such as the German S3 Guideline (https://www.leitlinienprogrammonkologie.de/fileadmin/user_upload/Downloads/Leitlinien/Melanom/Melanom_Version_3/LL_Melanom_Langversion_3.1.pdf). A closer look at the patients in whom PET/MRI led to a management change in our study reveals that, in 9/10 NSCLC patients (all stage III or IV), this change was due to detection of brain metastases on the MRI component. Contrary, in melanoma (five patients, all stage III or IV), reasons for management changes in our study were more balanced: brain metastases in 3/5 patients and liver metastases in 2/5 patients. In pancreatic adenocarcinoma and NETs (3 patients each), and colorectal cancer (one patient), management changes were exclusively due to liver metastases on the MRI component of PET/MRI. Notably, in the single patient with cervical cancer (referred as stage II) in whom PET/MRI lead to a management change, the higher soft-tissue contrast provided by morphological MRI enabled correct locoregional staging, ruling out bladder infiltration (a criterion for stage IV disease). Although more data are needed to confirm these findings, they nevertheless suggest that MRI protocols in the setting of PET/MRI may need to focus on different anatomic sites in different types of cancer, e.g., the brains in NSCLC and melanoma, the liver in melanoma, colorectal and pancreatic cancer, and NETs.

In the above scenarios, the use of PET/MRI instead of PET/CT obviates the need to perform additional, single-region MRI. Such considerations must be considered when looking at our *per-examination* costs, which show that PET/MRI is almost 50% more expensive than PET/CT. A clinically indicated addition of MRI to PET/CT may, depending on the body region and MRI protocol, result in a similar, or even higher overall cost than for PET/MRI, with the possible disadvantage of a prolonged time interval until treatment initiation. Furthermore, in our study, PET/MRI prevented inappropriate surgery in several cases (e.g., patient nos. 27, 106, 115, and 127 in Table 3), the cost for which exceeds the cost difference between PET/MRI and PET/CT. In one melanoma patient, treatment failure with ipilimumab was detected only by PET/MRI (patient no. 212 in Table 3 and Fig. 2), which was then discontinued and replaced by pembrolizumab—here, the cost for an inappropriate, additional cycle of ipilimumab (10,000–15,000 EUR, depending on body weight) would have exceeded the cost difference between PET/CT and PET/MRI by far.

Our study is limited by its monocentric design, which also affected our sample size. However, with 263 patients (330 examinations), we prospectively evaluated about twice as many patients as the largest study on this topic so far (Catalano et al., with 134 retrospectively included patients and 134 examinations) [17]. We enrolled only patients scheduled to undergo PET/CT for routine purposes, and thus, our sample reflects standard clinical care, in terms of cancer types and radiotracers, without any relevant selection bias. However, this strategy prevented us from including patients with untreated prostate cancer, who, in our institution, undergo [^68^Ga]Ga-PSMA-11-PET/MRI rather than PET/CT, and for whom participation in our study would have meant a purely study-related radiation exposure. Consequently, we cannot exclude the possibility that inclusion of prostate cancer patients would have influenced our study results—in view of the current literature, probably in favor of PET/MRI [21, 33, 34]. Furthermore, similar to the majority of studies on this topic [2, 8, 11, 17], we used a composite reference standard that relied chiefly on follow-up imaging, and, in a smaller number of patients, on biopsies, for verification of additional lesions detected exclusively by either PET/MRI or PET/CT, because it would have been unethical to perform strictly study-related invasive procedures. Our study design prevented us from performing an economic evaluation of changes in patient management due to the use of PET/MRI, as this would require (1) randomization of patients to either PET/MRI or PET/CT, (2) homogeneous cohorts in terms of cancer type(s) and predefined treatment trajectories, and (3) long-term follow-up including assessment of both clinical outcome and quality of life. Cost estimates—and in particular, absolute numbers—reflect the situation at our tertiary care center, and to a certain extent, trends within the country where our institution is located, but may not necessarily be applicable to other countries. Finally, our cost-effectiveness analyses focused on a direct comparison of the two hybrid imaging techniques, PET/MRI and PET/CT, but not on combinations with single-modality techniques, such as PET/CT combined with cranial MRI or PET/MRI combined with chest CT.

In conclusion, the results of our prospective study in a mixed oncologic patient population suggest that the choice of PET/MRI over PET/CT has implications for management in a non-negligible fraction of patients who routinely undergo PET/CT. In particular, patients with NSCLC and melanoma may benefit from PET/MRI, which detects brain and liver metastases that go undetected on PET/CT. Since the cost per PET/MRI examination is almost 50% higher than that of PET/CT, a histology-based triage of patients to either PET/MRI or PET/CT could be meaningful.
